# Factors associated with active commuting to school by bicycle from Bogotá, Colombia: The FUPRECOL study

**DOI:** 10.1186/s13052-016-0304-1

**Published:** 2016-11-15

**Authors:** Robinson Ramírez-Vélez, Cesar Augusto Beltrán, Jorge Enrique Correa-Bautista, Andres Vivas, Daniel Humberto Prieto-Benavidez, Javier Martínez-Torres, Héctor Reynaldo Triana-Reina, Emilio Villa-González, Antonio Garcia-Hermoso

**Affiliations:** 1 Centro de Estudios para la Medición de la Actividad Física (CEMA), Escuela de Medicina y Ciencias de la Salud, Universidad del Rosario, Bogotá, D.C Colombia; 2 Grupo de Ejercicio Fisico y Deportes, Vicerrectoria de Investigaciones, Universidad Manuela Beltrán, Bogotá, D.C Colombia; 3 Grupo GICAEDS, Programa de Cultura Física, Deporte y Recreación, Universidad Santo Tomás, Bogotá, D.C Colombia; 4 Department of Physical Culture, School of Health Sciences, National University of Chimborazo, Riobamba, Ecuador; 5 Department of Physical Education and Sport, PROFITH “PROmoting FITness and Health through physical activity” research group, School of Sport Sciences, University of Granada, Granada, Spain; 6 Laboratorio de Ciencias de la Actividad Física, el Deporte y la Salud, Universidad de Santiago de Chile, USACH, Santiago, Chile; 7 Facultad de Ciencias de la Actividad Física, Universidad San Sebastián, Santiago, Chile

**Keywords:** Physical activity, Factors associated, Active commuting, School

## Abstract

**Background:**

Active commuting to school (ACS) can contribute to daily physical activity (PA) levels in children and adolescents. The aim of the study was to analyze the characteristics of active commuting to and from school by bicycle and to identify the factors associated with the use of bicycles for active commuting to school based in a sample of schoolchildren in Bogotá, Colombia.

**Methods:**

A cross-sectional study was conducted in 8,057 children and adolescents. A self-reported questionnaire was used to measure frequency and mode of commuting to school and the time it took them to get there. Weight, height, and waist circumference measurements were obtained using standardized methods, and mothers and fathers self-reported their highest level of educational attainment and household level. Multivariate analyses using unordered multinomial logistic regression models were conducted in the main analysis.

**Results:**

21.9 % of the sample reported commuting by bicycle and 7.9 % reported commuting for more than 120 min. The multivariate logistic regression showed that boys, aged 9–12 years, and those whose parents had achieved higher levels of education (university/postgraduate) were the factors most strongly associated with a use bicycles as a means of active commuting to and from school.

**Conclusion:**

The findings of this study suggest that it’s necessary to promote ACS from childhood and to emphasize its use during the transition to adolescence and during adolescence itself in order to increase its continued use by students.

## Background

Current trends in promoting active and healthy lifestyles seek to implement strategies to mitigate problems such as excess weight in childhood and low levels of physical activity (PA) and sedentary behaviors among children [1, 2]. Current recommendations call for children and adolescents to engage in moderate to vigorous physical activity for 60 min every day [3]. One way to implement these PA recommendations is to walk or bicycle to school [4], activities that can be characterized as active commuting to school (ACS), understood as those activities in which the subject uses a dynamic means of transportation such as walking, bicycling, or another non-motorized means for commuting to and from school. In addition to its advantage as a strategy that is practical, accessible, and sustainable over the long term, using ACS provides an opportunity to increase children’s and adolescents’ daily levels of PA [5, 6].

Previous evidence has showed that engaging in ACS is an effective way to prevent excess weight and fatty tissue and diminishes some cardiovascular risk factors when these young people grow into adulthood [5–7]. In 2011, Larouche et al. [7] systematically reviewed 68 observational articles, demonstrating that ongoing bicycle commuting to and from school among 15 to 17-year-olds increased weekly levels of PA by 81.6 % and improved cardiovascular fitness by 12 %. In cross-sectional studies, Cooper et al. [8] and Larouche et al. [9] propose that the regular use of ACS to and from school is a strategy for implementing recommendations for moderate and vigorous PA. In addition, several longitudinal studies have demonstrated that ACS correlates with improved physical conditions tied to health, including muscular fitness, body composition, and aerobic fitness, which are recognized as significant markers of health among children and adolescents [10, 11]. In addition, lower levels of aerobic and muscular fitness among children are considered independent factors in relation to mental and physical well-being among adults. Thus, the inclusion of these two health indicators in school-based monitoring systems is clearly justified [12].

Although most schools incorporate physical education and the culture of ACS as important elements of their educational programs [13], the prevalence of ACS to and from school has decreased drastically in recent decades in countries such as the United States [14, 15], Australia [16], Canada [17], Spain [18], and the United Kingdom [19]. Unfortunately, there are few up-to-date studies on the prevalence of ACS among Latin Americans [20–22]. A Brazilian study by Kelly et al. [20] indicates a 70 % prevalence of ACS ranging from 10 to 20 min in duration. In Colombia, Arango et al. [21] reported that walking and bicycling were the means of ACS most used by students in the city of Montería, with values of 51.1 % and 15.2 %, respectively. This finding is similar to that described by Piñeros et al. [22] who found that 55.1 % of private school students and 41.1 % of public school students in Bogotá, Colombia reported that walking was the most commonly used form of ACS.

Several other factors have also been described as more or less influential determinants regarding the decision to commute actively, for example the students’ socioeconomic status, the characteristics of the natural environment, the educational level of parents, social support for active commuting, the distance between school and home, and the perceptions of parents and children regarding neighborhood characteristics [4, 7, 16, 23]. Sallis et al. [24] has produced research showing that school interventions that actively promote the participation of families and communities reinforce the use of ACS and can generate significant improvements in student health.

Despite the importance of ACS as a strategy to promote PA in keeping with recommendations proposed by the World Health Organization and other international bodies, and despite the high rates of physical inactivity and sedentary lifestyles among students in Bogotá, Colombia, few works have described these kinds of behaviors among this population [22, 25]. For this reason, our study has the following aims: a) to analyze the patterns of active commuting by bicycle to and from school among a sample of children and adolescents attending public schools in Bogotá, Colombia that participate in the FUPRECOL Study [26], and b) to identify factors associated with the use of bicycles for ACS among the same population.

## Methods

### Study design and sample

During the 2014–2015 school year, we conducted a cross-sectional study, a component of the FUPRECOL project [*in Spanish*, ASOCIACIÓN DE LA **FU**ERZA **PRE**NSIL CON MANIFESTACIONES DE RIESGO CARDIOVASCULAR TEMPRANAS EN NIÑOS Y ADOLESCENTES **COL**OMBIANOS (*Association for muscular strength with early manifestation of cardiovascular disease risk factors among Colombian children and adolescents*)]. Briefly, this study aimed to examine the relationships between physical fitness levels in children and adolescents with cardiometabolic risk factors and (un) healthy habits [27, 28]. The sample consisted of children and adolescents (boys *n* = 5,000 and girls *n* = 5,000) aged 9–17.9 years. There were no differences in the study key characteristics (i.e., age, sex distribution, body mass index [BMI], and use of bicycles) between the current study sample and the original FUPRECOL Study sample (*n* = 10,000, all *p* > 0.100). The children and adolescents were of low to middle socioeconomic status (SES, 1–3 defined by the Colombian government), enrolled in public elementary and high schools (grades 5 through 11), and from the capital district of Bogota in a municipality in the Cundinamarca Department in the Andean region. A convenience sample of volunteers was included and grouped by sex and age with 1-year increments (a total of 9 groups). Pregnant students and those with permanent physical, sensory, or intellectual handicaps were excluded as were those with non-communicable diseases such as diabetes type I or II, cardiovascular disease, diagnosed autoimmune conditions, or cancer. Also excluded were those known to abuse drugs or alcohol, and in general those who suffered from pathologies not directly related to nutrition such as congenital metabolic disorders, metabolic syndrome, morbid obesity, and psychiatric disorders such as anorexia or bulimia, among others. These exclusions were unbeknownst to participants and were made a posteriori in order to respect participants’ personal dignity and confidentiality. The school census of 546.000 students provided by the Capital District’s Secretary of Education in 2013 was used to calculate the size of the sample, using Schlesselmann’s equation [29] as applied to known samples, with α = 0,05 (95 % reliability). The estimated variance for overweight subjects (obesity/overweight) used for this population was 20 % based on the most recent National Survey of the Nutritional Situation, 2010 [30].

### Anthropometric measurements

Before the measurements and interviews were conducted, researchers and health and sports professionals held 10 theoretical/practical training sessions in order to standardize the process of morphological evaluation previously described in the FUPRECOL health and fitness battery [25–27]. To evaluate nutritional state, body mass were measured using a TANITA® model BF689 scale (Arlington Heights, IL 60005, USA) with a resolution of 100 kg. Height was measured using a portable SECA 206® (Hamburg, Germany) stadiometer with a range of 0–220 cm and 1 mm precision. Body Mass Index (BMI) was calculated in order to relate weight to height, using the formula proposed by *Quetelet* [BMI = weight (kg)/height (m)^2^]. Participants were subsequently classified as low weight (Z score = −2) normal weight (Z score > −2 to 1), or overweight (Z score > 1 to < 2) or obese (Z score = 2), based on the growth and development criteria proposed by Cole et al. [31] Waist circumference (WC) was measured using the anatomical referents and protocols described by the World Health Organization [32] using a stretch‐resistant metal measuring tape at the end of several consecutive natural breaths, midpoint between the top of the iliac crest and the lower margin of the last palpable rib in the mid axillary line with the subject standing upright during the measurement, with arms relaxed at the side, feet evenly spread apart and body weight evenly distributed. WC was considered to be at the level of abdominal obesity (unhealthy or at risk) when this measurement was above the 90th percentile of the referents suggested by Ferranti et al. [33] taking sex and age into account to establish diagnostic criteria for metabolic syndrome in subjects under 18 years of age.

### Determining the means of commuting to school

Self-perceived commuting to school as well as cut-off point, were assessed by metodology of the Health Behaviour in School-Aged Children (HBSC) questionnaire [2]. The method of commuting to school was elicited by asking the question: *“Have you used a bicycle to get to school and get back home?* Youths were asked to quantify their commuting to school in the previous week, recording the data from Monday to Friday. Responses were categorized as active: “Yes” (if they commuted by bicycle) and passive: “No” (if they commuted in a motor vehicle). Researchers also sought to determine how much time was spent commuting to and from school by either bicycle or motor vehicle, asking the question: *How long did it take you to get to school and return home those days in minutes and hours?”* Responses to this question were categorized such as: 0–59 min per day; 60–119 min per day; or ≥ 120 min per day.

### Factors associated with the means of getting to school

The following variables were analyzed as associated factors for this study: i) sex: (male/female); ii) age group: (child [9–12 years]/adolescent [13–17 years]); iii) abdominal obesity: (healthy/at risk); iv) BMI classification: (low weight/normal or healthy weight/overweight or obese); v) highest educational level attained by mother/father: (not reported/primary or secondary/technical/technological/university/postgraduate); and vi) composition of household: (lives with father/lives with mother/lives with both parents/lies with grandparents or other relatives). Surveys were distributed to groups of 20 to 50 participating students in classrooms with at least two qualified researchers present for the purpose of maintaining privacy and ensuring voluntary participation. Before the surveys were distributed and the nutritional measurements made, the necessary requirements for filling out the surveys correctly were explained with an emphasis on the need to read the questions carefully, to respond honesty, and to maintain anonymity at all times.

### Ethical aspects

The Review Committee for Research on Human Subjects at the University of Rosario [Code N° CEI-ABN026-000262] approved all of the study procedures. A comprehensive verbal description of the nature and purpose of the study and its experimental risks was given to the participants and their parents/guardians. Written informed consent was obtained from parents and subjects before participation in the study. The protocol was in accordance with the latest revision of the Declaration of Helsinki and current Colombian laws governing clinical research on human subjects (Resolution 008430/1993 Ministry of health).

### Statistical analysis

Continuous values were expressed in terms of mean standard deviation and frequency of ordinal variables. Tests of homogeneity and variance were applied (*analysis of variance*; ANOVA) to study the differences between continuous variables and the *chi* squared (*X* ^2^) test of categorical variables. An exploratory analysis was subsequently carried out to determine the percentage distribution for each of the associated factors and means of commuting. Finally, a binary logistical regression was applied to determine the association between the factors studied with the means of commuting as the event of interest. The regression models were adjusted for gender, age, nutritional status and center. These analyses were carried out using version 20 of the software program *Statistical Package for Social Science®* (SPSS; Chicago, IL, United States), and a value of *p* < 0.05 was considered significant.

## Results

The sample consisted of 8,057 students attending 28 public schools in the city of Bogotá, Colombia (response rate 80 %). Among this general population, 55.6 % were female, with an average age of 12.9 ± 2.3 years, body weight 45.1 ± 11.4 kg, height 149.1 ± 10.1 cm and BMI 20.0 ± 3.5 kg/m2. The ANOVA analysis showed that males had higher age, weight, height, and waist circumference than did females (*p* < .001), while females had higher mean BMI (*p* < .001) and a greater proportion of participants of mean weight (*p* = 0.008) (Table 1).

**Table 1:** General characteristics *(n* = *8057)*

Characteristics	Female *n = 4481*	Male *n = 3579*	*p*
*Anthropometry* (mean ± SD)^*a*^			
Age (years)	12.9 ± 2.3	13.0 ± 2.4	<0.001
Weight (kg)	45.1 ± 11.4	46.1 ± 13.0	<0.001
Height (cm)	149.1 ± 10.1	152.7 ± 14.1	<0.001
BMI (kg/m2)	20.0 ± 3.5	19.4 ± 3.3	<0.001
Nutritional status (%)^b^			
Underweight	779 (58.3 %)	557 (41.7 %)	0.662
Healthy	2283 (50.2 %)	2262 (49.8 %)	0.008
Overweight/Obesity	1419 (65.1 %)	760 (34.9 %)	0.501
Waist circumference (cm)	64.6 ± 8.0	66.1 ± 8.1	<0.001
Abdominal obesity (%)^b^	239 (52.0 %)	221 (48.0 %)	0.106

*BMI* body mass index

^a^One-way ANOVA differences

^b^Differences (*X*2)

**Fig. 1:**
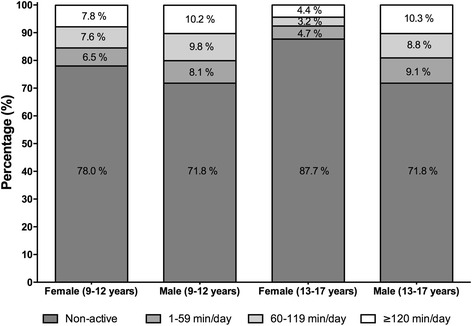
Travel time for active commuting to and from school by bicycle

Of the students surveyed, 21.9 % actively commuted to school using a bicycle (Fig. 1). This study found that there were differences by sex and age in the proportion of students using ACS by bicycle to get to school and back home. Fewer adolescent girls 13–17 years old reported bicycle use to get to school compared to 9-12-year-old girls (≈10 %), while older boys did not show a reduction of bicycle use for this purpose.

Of the students who commuted to school actively (21.9 %), 6.9 % showed time values of 1–59 min per day to commute, while 7.9 % took more than 120 min per day. There were no differences between the sexes (*p* = 0.180), but abnormally obese students had greater representation in the group that invested the lesser time value of 1–59 min per day. One interesting data point is that commuting to school by bicycle decreases as age increases, even though there were no differences in the time taken for active commuting. No differences were observed with respect to the other factors analyzed such as the highest level of educational attainment of the father or mother, household composition, or anthropometric variables (Table 2).

**Table 2:** Active commuting to school according to related factors in schoolchildren from Bogotá, Colombia

Factors	Non-active		Active^b^					
			1–59 min/día		60–119 min/día		>120 min/día	
	n	%	n	%	n	%	n	%
Total	6298	78.1	560	6.9	566	7.0	636	7.9
Gender								
Male	3729	83.2	248	5.5	236	5.3	268	6.0
Female	2569	71.8	312	8.7	330	9.2	368	10.3
Age								
Children (9–12 años)	2724	75.4	261	7.2	310	8.6	319	8.8
Adolescent (13–17 años)	3574	80.4	299	6.7	256	5.8	317	7.1
Waist circumference								
Abdominal obesity	357	77.6	36	7.8	32	7.0	35	7.6
Healthy	5941	78.2	524	6.9^a^	534	7.0	601	7.9
Nutritional status								
Underweight	1047	78.4	103	7.7	92	6.9	94	7.0
Healthy	3523	77.5	313	6.9	337	7.4	372	8.2
Overweight/Obesity	1728	79.3	144	6.6	137	6.3	170	7.8
Father’s academic level								
No report	849	76.1	90	8.1	78	7.0	98	8.8
Elementary or high school	4574	79.1	382	6.6	403	7.0	424	7.3
Technician or technologist	531	77.1	48	7.0	48	7.0	62	9.0
University or post-graduate	344	72.7	40	8.5	37	7.8	52	11.0
Mather’s academic level								
No report	612	75.4	63	7.8	59	7.3	78	9.6
Elementary or high school	4641	79.0	390	6.6	405	6.9	439	7.5
Technician or technologist	681	78.7	60	6.9	47	5.4	77	8.9
University or post-graduate	364	71.7	47	9.3	55	10.8	42	8.3
Family structure								
Parents	3287	77.7	299	7.1	307	7.3	339	8.0
Mother	2099	79.3	180	6.8	173	6.5	196	7.4
Father	287	80.8	26	7.3	17	4.8	25	7.0
Grandparents	152	75.2	14	6.9	14	6.9	22	10.9
Others	224	76.7	20	6.8	21	7.2	27	9.2
Anthropometry	Mean	SD	Mean	SD	Mean	SD	Mean	SD
Waist circumference (cm)	65.4	8.1	65.5	8.1	64.4	7.5	65.2	8.2
Mass (kg)	45.9	12.1	45.2	12.2	43.2	11.8	44.8	12.9
Heigth (cm)	150.9	12	151.2	12.2	148.9	13.3	150.1	13.2
BMI (kg/m2)	19.9	3.4	19.5	3.3	19.2	3.1	19.5	3.4

*BMI* body mass index, *SD* standard deviation,

^a^Differences (*X*2) *p* < 0.05

^b^Use of bicycle

**Fig. 2:**
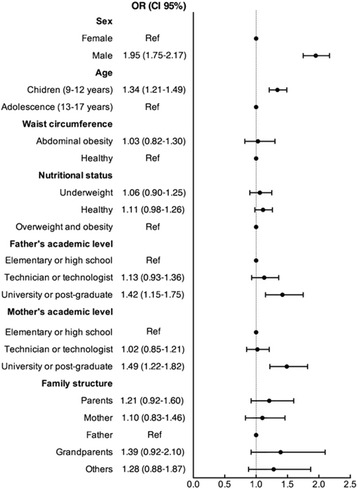
Factors associated with active commuting to school by bicycle by students in Bogotá, Colombia

Associations between the factors analyzed with respect to active commuting to school by bicycle are shown in Fig. 2. A simple logistic regression shows a greater probability of using a bicycle as a means of active commuting to school among males [OR 1.95 (CI 95 % 1.75–2.17)], among youth 9–12 years old [OR 1.34 (IC 95 % 1.21–1.49)], and those whose father or mother reported academic achievement at the university or postgraduate level [father OR 1.42 (CI 95 % 1.15–1.75)/mother OR 1.49 (CI 95 % 1.22–1.82)] also had a greater likelihood of using a bicycle as a means of active commuting to school.

## Discussion

This study analyzes the prevalence of active commuting to and from school by bicycle and the factors that are associated with this active commuting among a sample of students in the Capital District of Bogotá, Colombia. The percentage of students who commuted by bicycle among the total sample of the study was 21.9 %. Among the factors analyzed, the most determinative factor affecting the behavior of commuting to school by bicycle was that students’ fathers and mothers reported a high level of educational achievement (university/postgraduate).

As has previously been determined in the scientific literature through different longitudinal studies, active commuting to school by bicycle can improve the physical condition (primarily cardiovascular) of young people, diminishing cardiovascular risk among them and generating a positive effect on their body composition, which is directly related to health [18, 34, 35]. These variables have been discussed with respect to other means of active commuting such as walking [36]. In this study, the total percentage of active commuting by bicycle was (21.9 %), a greater percentage than that reported in another study that analyzed the prevalence of the same behavior among Colombians 11–18 years of age in the city of Montería (15.2 %) [21]. The percentages shown in most studies in other countries are lower than in this study, specifically in Spain (1 %) [37], England (8 %) [19], and Canada (3 %) [38], primarily due to the development of urban infrastructure and the difficulties in using bicycles due to the characteristics of cityscapes in those countries.

As with physical activity, the scientific literature indicates dissimilar results with respect to ACS and gender differences. The AVENA (Feeding and Assessment of Nutritional Status of Spanish Adolescents) study [18] of Spanish adolescents showed that unlike the results of our study, ACS is more common among females, although the means of commuting to and from school referred to was walking. A study of Danish adolescents showed that a greater proportion of males used bicycles for commuting [11]. These results seem to suggest that gender differences regarding ACS may depend on the specific means of commuting in question. Regarding age, this study shows that the proportion of students who used bicycles to get to and from school decreased among adolescent girls between 13 and 17 years old (≈10 %) compared to girls from 9 to 12 years old, while the percentage of boys using bicycles did not change as they matured. The AVENA study confirms reduced ACS over time. In Canada, a study of active commuting among adolescents compared three age groups (9, 13 and 16 years of age), and concluded that active commuting diminished as children’s age increased [39]. Although previous evidence demonstrates that patterns of active commuting by bicycle change as age increases from childhood to adolescence, it seems that there weren’t differences when the sexes were compared [18]. These patterns are similar in childhood, but the use of bicycles increases as children become adolescents (unlike in the case of walking, which decreases). This increased use of bicycles could be primarily due to parents’ increased perception of safety on the routes that their sons and daughters take [40, 41] and the students’ increasingly independent mobility. Another reason for the increased use of bicycles at these ages is the greater distance between households and schools [42] that usually characterizes Secondary (as opposed to Primary) Education in Bogotá. Unfortunately, we were unable to measure the distance between households and schools in the present study, although we did quantify the minutes spent to make each trip, which did not point to any significant differences between the sexes. Previous studies have also quantified the time that children and adolescents take to get to school [38, 43].

Several studies have analyzed the factors that influence young people’s means of commuting to and from school. The most commonly reported factors and those that are apparently reliable predictors of active commuting (usually by foot and by bicycle) are the distance between the household and the school, and family-driven factors such as parents’ evaluation of public safety, fear of traffic en route, and convenience [44]. In this study the factor shown to be most determinative for active commuting to school was the level of educational achievement of the parent or parents; it was found that a higher level of educational achievement (university or postgraduate) correlated with a greater probability of children’s active commuting to school by bicycle. A systematic review concluded that socioeconomic status had a significant influence on active commuting to school [40], given that students from lower socioeconomic strata were more likely to be active than those from higher socioeconomic strata. The same review also indicated that children of parents reporting lower levels of educational achievement were also more likely to commute to school walking or by bicycle. In most cases, this association has to do with car ownership and the fact that these parents also considered the routes that their children take to school to be safer. These findings contradict our results, in which it was found that a higher level of educational achievement by fathers and mothers was associated with a greater likelihood that their children would commute to school by bicycle. This disparity may be explainable by the fact that all of the students belonged to what are considered low socioeconomic strata, and were therefore more likely to commute to school by active means, independent of the level of educational achievement of their fathers and mothers. It should also be taken into account that these kinds of active behaviors have multifactorial bases, since they can also be associated with personal, family, or environmental factors, some of which were not considered in this study.

On another note, active commuting by bicycle was more frequent among students suffering from abdominal obesity among the group that invested 1–59 min per day compared with other groups that invested more time commuting, which accords with the results of a previous study that found a negative association between active commuting to school by bicycle and overweightedness of students in Rotterdam and Kristiansand [45]. Nevertheless, we caution against drawing conclusions since it is not yet clear from the international literature that there is an association between commuting to school by bicycle and student weight [7].

With this understanding, it can be concluded that a higher level of educational achievement by father and mother is associated with a greater probability of active commuting to and from school by bicycle, which cannot be said with respect to the other factors analyzed. In addition, an age of 6–13 years and male gender correlate to greater use of active commuting by bicycle. Taking the findings of this study into consideration, it seems necessary to promote ACS from an early age, putting greater emphasis on the transition to adolescence and among adolescents themselves in order to increase their daily levels of PA. Nonetheless, there is a need for longitudinal studies to measure other factors whose influence is unclear and that may complicate the interpretation of these results.

The primary limitations of this study are inherent to its cross-sectional nature and the type of sample used. It would be important to increase the population sample studied by including different age groups or by expanding the survey to private schools. The reason for selecting a sample of students 9–17 years old was the variability of PA habits that can be found among this age group. The works of Janz et al. [46] and Castillo-Garzón et al. [47] show that a low level of PA in childhood is associated with a greater risk of cardiometabolic disease in adulthood. In addition, a lower level of PA in childhood [48] is considered an independent factor influencing mental and physical well-being in adulthood [49]. For this reason, the inclusion of this health indicator in school-based epidemiological monitoring systems is clearly justified [50]. In addition, the self-reporting of ACS may either underestimate or overestimate the levels of student PA. That is a main limitation since the responses cannot be verified. Some questions in the ACS questionnaire may have been misunderstood either intentionally or inadvertently by some participants. However, intentional misreporting was probably minimized by the fact that the questionnaires were filled-in anonymously, and questions in the HBSC questionnaire have shown good reliability and validity in children/youth population [2]. However, such limitations do not compromise the results obtained when validating our results. In this line, Phillips et al. [51] and Leblanc et al. [52] recently reported, however, these findings are useful as a dependent variable or to determine some of the components of physical condition without measuring the frequency of the behavior. These researchers found that self-reported PA is a predictor of general health and fitness levels, suggesting that the self-reporting of ACS could also be an indirect measurement of the level of PA among young students. It is worth pointing out that other variables previously associated with ACS were not evaluated, including the distance from the household to the school, the objective levels of PA, the particular cityscape and environment proximate to the school, socioeconomic and social factors, and physical conditions that could interfere to some extent with the results of this investigation. Despite these limitations, there is no reason to believe that the described relations are exclusively relevant to the population represented in our sample, since the results of other Colombian and international studies accord with the results of this study [10, 18, 19, 21, 37, 38] rather than undermining them.

## Conclusion

In conclusion, this study shows that one out of every five of the students evaluated reported using a bicycle for commuting and about 7 % took longer than 120 min to get to or from school. It was observed that males, adolescents from 9 to 12 years of age, and students whose parents reported a higher level of educational achievement, specifically university/postgraduate, were more likely to actively commute to school by bicycle. The findings of this study suggest that it is necessary to promote ACS from a young age, putting particular emphasis on the transition to adolescence and on adolescents themselves in order to increase their daily levels of PA.

## Abbreviations

ACS: Active commuting to school
ANOVA: *Analysis of variance*
AVENA study: Feeding and assessment of nutritional status of spanish adolescents
BMI: Body mass index
FUPRECOL project [*in Spanish*]: ASOCIACIÓN DE LA **FU**ERZA **PRE**NSIL CON MANIFESTACIONES DE RIESGO CARDIOVASCULAR TEMPRANAS EN NIÑOS Y ADOLESCENTES **COL**OMBIANOS
PA: physical activity
WC: Waist circumference
